# Numerical Magnitude Processing in Deaf Adolescents and Its Contribution to Arithmetical Ability

**DOI:** 10.3389/fpsyg.2021.584183

**Published:** 2021-03-25

**Authors:** Lilan Chen, Yan Wang, Hongbo Wen

**Affiliations:** ^1^School of Psychology, Hainan Normal University, Haikou, China; ^2^Faculty of Education, Beijing Normal University, Beijing, China; ^3^Collaborative Innovation Center of Assessment Toward Basic Education Quality, Beijing Normal University, Beijing, China

**Keywords:** numerical magnitude representation, arithmetic computation, congenital deafness, acquired deafness, mathematical cognition

## Abstract

Although most deaf individuals could use sign language or sign/spoken language mix, hearing loss would still affect their language acquisition. Compensatory plasticity holds that the lack of auditory stimulation experienced by deaf individuals, such as congenital deafness, can be met by enhancements in visual cognition. And the studies of hearing individuals have showed that visual form perception is the cognitive mechanism that could explain the association between numerical magnitude processing and arithmetic computation. Therefore, we examined numerical magnitude processing and its contribution to arithmetical ability in deaf adolescents, and explored the differences between the congenital and acquired deafness. 112 deaf adolescents (58 congenital deafness) and 58 hearing adolescents performed a series of cognitive and mathematical tests, and it was found there was no significant differences between the congenital group and the hearing group, but congenital group outperformed acquired group in numerical magnitude processing (reaction time) and arithmetic computation. It was also found there was a close association between numerical magnitude processing and arithmetic computation in all deaf adolescents, and after controlling for the demographic variables (age, gender, onset of hearing loss) and general cognitive abilities (non-verbal IQ, processing speed, reading comprehension), numerical magnitude processing could predict arithmetic computation in all deaf adolescents but not in congenital group. The role of numerical magnitude processing (symbolic and non-symbolic) in deaf adolescents' mathematical performance should be paid attention in the training of arithmetical ability.

## Introduction

Mathematical knowledge and ability play an important role in the successes of our social life (Ritchie and Bates, 2013), but most deaf individuals have some difficulty in acquisition of arithmetical skills even if they have the approximately same level of non-verbal intelligence as hearing peers (Braden, 1994; Moreno, 2000). Many studies have shown the close association between numerical magnitude processing and mathematical ability (LeFevre et al., 2010; De Smedt et al., 2013; Sasanguie et al., 2013; Fazio et al., 2014; Linsen et al., 2015), although few studies on the arithmetical abilities of deaf individuals (Masataka, 2006; Andin et al., 2014, 2020). It has been found in many studies that the poorer performance of deaf individuals in mathematics has generally been associated with their reduced language abilities (Kelly and Gaustad, 2007; Wu et al., 2013; Huber et al., 2014; Vitova et al., 2014).

Although most deaf individuals could use sign language or sign/spoken language mix, hearing loss would still affect their language acquisition (Kennedy et al., 2006; Elizabeth et al., 2016). Individuals who lose the hearing before acquiring speech and language, such as those with congenital deafness, are at a much greater disadvantage than those with acquired deafness in the interdependent language processes such as: thought development, concepts in number, measurement, operations, problem solving, and so on (Pagliaro and Kritzer, 2013; Pénicaud et al., 2013).

According to the Triple Code Model (TCM; Dehaene, 1992), a model of numerical processing proposes that numbers are represented in three codes: analog magnitude representation, auditory verbal representation, and visual Arabic representation. Dehaene and Cohen (1995, 1997) proposed two major transcoding paths between the three representational codes: a direct a semantic route that transcodes written numerals to auditory verbal to guide retrieval of rote knowledge of arithmetic facts without semantic mediation, and an indirect semantic route specialized for quantitative processing that manipulates analog magnitude representations by manipulating visual Arabic representations. Neuropsychological studies found some patients demonstrated impairment in tasks involving verbal representations of number, but could perform tasks involving non-verbal representations of number (Cipolotti and Butterworth, 1995; Cohen et al., 2000).

Although the time of onset of hearing loss is known to be an important factor influencing the academic performance of deaf individuals (Moores, 1985; Paul and Quigley, 1990; Liu, 2013), little research has focused on the arithmetical abilities of individuals with congenital or acquired deafness. Moreover, many studies focused on gender differences and mathematical performance in hearing population found male advantage in mathematics (Burton and Lewis, 1996; Gallagher et al., 2000; Perie et al., 2005; Liu and Wilson, 2009), while some showed that girls outperformed boys in numerical magnitude processing (Wei et al., 2012) and arithmetic computation (Linn and Hyde, 1989; Willingham and Cole, 1997; Wei et al., 2012), others revealed no gender differences in children's mathematical ability (Kersey et al., 2018; Zhang et al., 2020).

Therefore, the first aim of the present study is to investigate the presence of differences among the performance of hearing adolescents, adolescents with congenital and acquired deafness in the tasks of numerical magnitude comparison and arithmetic computation; a second aim is to explore the gender differences in numerical magnitude processing and arithmetic computation of deaf adolescents. And the third aim is to examine the predictive role of numerical magnitude processing on arithmetical abilities of deaf adolescents.

### Numerical Magnitude Processing and Mathematical Ability

Numerical magnitude processing, as the mental manipulation of quantitative information of either symbolic numbers (e.g., Arabic digits) or non-symbolic quantities (e.g., dot arrays) (Turconi et al., 2004; Tudusciuc and Nieder, 2007) has been found important for successful mathematical development (e.g., Butterworth et al., 2011, for a review) and positively associated with mathematical performance of the hearing individuals (Sasanguie et al., 2013; Fazio et al., 2014; Schneider et al., 2017). Further research by Zhang et al. (2016) found that numerical magnitude processing was the independent predictor of arithmetical computation but not mathematical reasoning for hearing children. Butterworth (2005) claimed that numerical magnitude processing was one of the reasons for dyscalculic difficulties in arithmetic.

For deaf individuals, it has also been found that ANS (approximate number system) acuity (non-symbolic magnitude processing) is significantly associated with mathematical performance, and less acuity in the ANS, compared to hearing peers, may be the reason for their delays in mathematics achievement (Bull et al., 2006, 2018). Some studies showed significant differences between deaf and hearing individuals in response times for numerical magnitude comparison (Epstein et al., 1995; Marschark et al., 2003). However, other researchers found no significant differences between deaf and hearing individuals in their number representation processes (Zarfaty et al., 2004; Arfé et al., 2011; Barbosa, 2013). Whether numerical magnitude processing (symbolic, non-symbolic) is a predictor of the arithmetical ability of the deaf population or not still needs to be verified.

### Numerical Magnitude Processing in Hearing-Impaired and Deaf Individuals

Numerical magnitude processing, or numerical magnitude representation process, in hearing-impaired and deaf individuals has been analyzed in both children and adults. Zarfaty et al. (2004) compared 3- and 4-year-old deaf and hearing children's performance in number representation tasks and found out the better performance of deaf children in the spatial task and no difference from hearing counterparts in the temporal tasks. Barbosa (2013) conducted a similar study with Brazilian deaf children aged 5–6 years, and found out the young deaf children's number representation ability was as good as that of hearing children, which supported the previous research findings by Zarfaty et al. (2004). Arfé et al. (2011) investigated number representation ability of deaf primary school children with cochlear implants in a digit comparison task and an analogic comparison task, and also found out the better performance of deaf children in the analogic task and no difference from hearing children in the digit comparison task. All of these studies, on symbolic magnitude processing, confirmed that deaf children present the same abilities in number representation as their hearing peers.

Bull et al. (2005) investigated deaf adults' performance on a magnitude comparison task, and found that deaf participants performed more slowly than hearing participants in making comparative judgments. However, there was no substantial difference in the basic numerical magnitude processing capacity between the deaf and age-matched hearing peers. Rodríguez-Santos et al. (2014) explored deaf and hard-of-hearing children's numerical magnitude representation process by means of symbolic (Arabic digits) and non-symbolic (dot constellations) magnitude comparison tasks, and found out slower reaction times of deaf participants in the symbolic but not non-symbolic task, which was believed to the delay that deaf individuals experienced in accessing representations from symbolic codes. Bull et al. (2018) also found that children with hearing loss had poorer numerical discrimination skills and less acuity in the ANS (non-symbolic magnitude processing) compared to hearing peers. As we can see, previous studies have rarely examined both the symbolic and non-symbolic magnitude processing of deaf individuals at the same time, except for Rodríguez-Santos et al. (2014), and diverged in whether they have the similar numerical magnitude representation to their hearing peers.

### The Present Study

As language abilities can support mathematical performance in deaf individuals (Kelly and Gaustad, 2007; Andin et al., 2014; Huber et al., 2014; Vitova et al., 2014) and some studies have showed a link between sign language skills and reading ability in deaf individuals (Mayberry et al., 2011; Rudner et al., 2012), reading comprehension has been used as a task to evaluate the linguistic performance for deaf adolescents, and as a control variable in this study. And according to previous studies, the general cognitive abilities (i.e., non-verbal IQ, processing speed) of deaf students affect their mathematical performance (Chen et al., 2019; Chen and Wang, 2020), so we also take the non-verbal IQ and processing speed as the control variables.

The aim of the present study was to examine numerical magnitude processing and its contribution to arithmetical ability in deaf adolescents. Firstly, according to the TCM, indirect semantic route supports the non-verbal numerical magnitude processing that manipulates analog magnitude representations by manipulating visual Arabic representations. Compared to the acquired deafness, individuals with congenital deafness may be more dependent on this non-verbal numerical magnitude processing due to auditory deprivation. Therefore, we want to explore whether there are differences across groups of congenital and acquired deafness in numerical magnitude processing and arithmetical ability.

Secondly, the researches on whether there are gender differences in mathematical performance of hearing individuals are still controversial, so we want to explore the gender differences in numerical magnitude processing and arithmetic computation of deaf adolescents. Thirdly, in view of the importance of mathematical ability and the lag of deaf children in arithmetic (Traxler, 2000; Swanwick et al., 2005; Gottardis et al., 2011), against the background of the found associations between numerical magnitude processing and arithmetical ability of hearing individuals (Fazio et al., 2014; Zhang et al., 2016; Schneider et al., 2017) and no significant differences between deaf and hearing individuals in their number representation processes (Zarfaty et al., 2004; Arfé et al., 2011; Barbosa, 2013), we also aimed to examine whether numerical magnitude processing (symbolic, non-symbolic) is a predictor of the arithmetical ability of the deaf adolescents.

## Methods

### Participants

The study included 58 congenital deaf adolescents [M_age_ = 184.36 (107–227) ± 28.12 months; 29 girls; M_unaided PTA loss in better ear_ = 98.54 ± 16.45 dB, 60–120 dB; Note PTA means Pure Tone Average; In amplification: 21 use of hearing aids, 10 use of cochlear implants, 31 no use of hearing aids and cochlear implants; Mode of family communication: 35 in Mandarin sign/spoken language mix, 16 in spoken Mandarin, seven in Mandarin sign language], 54 acquired deaf adolescents[M_age_ = 188.44 (99–231) ± 26.48 months; 27 girls; M_unaided PTA loss in better ear_ = 99.29 ± 12.72 dB, 75–110 dB; In amplification: 26 use of hearing aids, eight use of cochlear implants, 22 no use of hearing aids and cochlear implants; Mode of family communication: 47 in Mandarin sign/spoken language mix, six in spoken Mandarin, one in Mandarin sign language], and 58 hearing adolescents [M_age_ = 166.34 (98–187) ± 21.75] months; 27 girls. Deaf participants were recruited from the special education schools in the Haikou municipality of Hainan Province in China with moderate to severe hearing impairment (60–120 dB). All participants had normal or corrected-to-normal vision. Adolescents with congenital deafness, who were born with deafness, were assigned to the congenital group, adolescents with acquired deafness, whose hearing impairment was not present at birth but developed sometimes during life, were assigned to the acquired group. The congenital and acquired groups matched in age, gender, hearing loss, and intelligence; all the groups (including hearing group) matched in intelligence. The university's institutional review board approved the study. Participants' and their parents' consents were obtained prior to classroom-based testing.

### Measures

#### Non-verbal IQ

The non-verbal matrices task, which was adapted from Raven's Progressive Matrices (Raven, 2000), was used to assess non-verbal IQ. It is a simplified version of Raven's Progressive Matrices that only had two candidate answers for each question, instead of 4–6 choices in the original version. Due to time constraints, the task was shortened to 80 items, 44 of which came from Standard Progressive Matrices (12 from the first set and eight from each of the other four sets) and 36 from Advanced Progressive Matrices. In the test, a large figure with a missing segment appeared in the center of the computer screen, and there were two options below. Participants were asked to identify the missing segment according to the rules underlying the figure, and pressed the “Q” key when the missing segment was on the left or the “P” key when it was on the right.

#### Processing Speed

A simple reaction time task was used to measure the processing speed [cf., Butterworth's (2003) “Dyscalculia Screener,” which included a reaction time task]. Each trial presented a fixation “+” in the center of the black computer screen, and a white dot appeared at the 30 degree angle randomly on the left or right side of the fixation “+.” Participants were asked to press the “Q” key when the white dot appeared on the left or the “P” key when the white dot appeared on the right. There were 30 trials in the test, of which 15 were white dots on the left and 15 were white dots on the right side of the fixation “+.” The dots were randomly presented, and the interval between responses and stimuli was varied randomly between 1000 and 2000 ms.

#### Reading Comprehension

The sentence completion task, which was adapted from Siegel and Ryan (1988), was used to measure reading comprehension (Elbeheri et al., 2011; Träff et al., 2018; Cui et al., 2019). Materials for the task were selected from the test materials used in primary and middle schools in China (from first to ninth grade). On the test, a sentence was presented in the center of the computer screen with a word missing and there were two options below. Participants were asked to choose a word from the options to complete the sentence and press the “Q” key if the correct answer was on the left, or press the “P” key if the correct answer was on the right. There were 120 problems on the test, ordered from easy to difficult, and the time interval for each problem was 1000 ms.

#### Numerical Magnitude Comparison

##### Symbolic Magnitude Comparison

A classic numerical magnitude comparison task, which was adapted from Zhou et al. (2007), used a Stroop-like paradigm to measure the ability to compare numerical values of numbers that varied in physical size (1:2 size ratio). In this task, participants had to indicate the numerically larger of two simultaneously presented Arabic digits (ranging from 2 to 9), one displayed on the left and the other on the right side of the computer screen in random orders, ignoring the differences in physical size. The position of the largest number was counterbalanced. There were 84 trials, and the stimulus interval was 1000 ms.

##### Non-symbolic Magnitude Comparison

The non-symbolic magnitude comparison task, which was used to assess approximate number sense (ANS) (e.g., Wei et al., 2012; Zhou et al., 2015), was divided into three sessions, with 40 trials in each session, and participants were required to complete all 120 trials. In this task, participants had to indicate the larger of two simultaneously presented dot arrays with different sizes and numbers, one displayed on the left and the other on the right side of the computer screen, ignoring all visual properties, such as total surface area, envelope area, diameter, and circumference. The dot arrays were created following a common procedure to control for continuous quantities in non-symbolic numerical discrimination (e.g., Halberda et al., 2008; Agrillo et al., 2013). The number of dots in each dot array varied from 5 to 32. The position of the largest numerosity was counterbalanced. The presentation time of each trial was 200 ms, and the interval time was 840 ms.

#### Arithmetic Computation

##### Simple Subtraction

The simple subtraction task, which consisted of 92 problems, was the reversed operation to single-digit addition. For each trial, a subtraction problem (e.g., 17–9) of <20 was presented at the top of the computer screen, and two candidate answers were presented on the bottom. The largest minuend of the problem was 18, and the smallest one was 2. The differences between two operands were always single-digit numbers, so the answer ranged from 2 to 9. The false candidate answer deviated from the true answer by plus or minus 1 to 3 (i.e., ±1, ±2, or ±3). Participants were asked to press the “Q” key if the true answer was on the left or press the “P” key if it was on the right. This was a time-limited (2 min) task, and the interval time of each trial (problem) was 1000 ms.

##### Complex Subtraction

The complex subtraction task, which consisted of 95 problems, included double-digit numbers for both operands. For each trial, a subtraction problem (e.g., 82–37) of <100 was presented at the top of the computer screen, and two candidate answers were presented on the bottom. Borrowing was required for most problems. The differences between the false answers and the true answers were 1 or 10. The task was limited to 2 min, and the interval time of each problem was 1000 ms.

### Procedure

All participants were tested at their own school during regular school hours and all tasks were computerized using the E-prime 2.0 software and were all administered using a 15 inch laptop individually in a quiet room. The experimenters, the teachers of the participants in the Department of Deaf, who were proficient in sign language and familiar with the specific situation of the participants, explained the instructions with slides and sign language and participants were instructed to perform both accurately and quickly by pressing the “Q” or “P” keys on a computer keyboard. Before the formal testing started, there was a practice session and feedback: When the item was correctly answered, the computer screen read “Correct! Can you go faster?” When participants answered incorrectly, the screen read “It is wrong. Try again.” Each trial started with a 200 ms fixation cross in the center of the computer screen. After 1000 ms the stimuli appeared and remained visible until response, except for the non-symbolic magnitude comparison task where the stimuli disappeared after 840 ms, in order to avoid counting. Accuracy (ACC) and RT (in milliseconds) were recorded for processing speed and numerical magnitude comparison tasks. Answers and reaction times were recorded by the laptop.

In order to control for the effect of guessing, the adjusted score was used in the tests such as non-verbal IQ, reading comprehension and arithmetic computation (simple and complex subtraction). It was calculated by subtracting the number of incorrect responses from the number of correct responses following the Guilford correction formula “S = R – W/(n – 1)” (S: the adjusted number of items that the participants can actually perform without the aid of chance. R: the number of right responses, W: the number of wrong responses. n: the number of alternative responses to each item) (Guilford, 1936). This correction procedure has been utilized recently in studies of mathematical cognition (Cirino, 2011; Zhou et al., 2015; Cui et al., 2019).

### Statistical Analyses

The statistical analyses were conducted using the Statistical Package for the Social Sciences (SPSS, version 25.0). Descriptive statistics were computed for demographic data and all study variables. One-way analyses of variance (ANOVAs) and LSD *post-hoc* comparisons were carried out to compare the differences in all the measures on the study groups. The repeated measurement analyses of variance (ANOVAs), with the group (congenital deaf adolescents, acquired deaf adolescents, hearing adolescents) and gender as between-subject factors and mathematical tasks as within-subject factors, were conducted to analyze group differences for accuracy and reaction times in the two numerical magnitude comparison tasks and the scores in arithmetic computation tasks. In order to control the effect of general cognitive abilities (e.g., reading comprehension) on mathematical tests, we used non-verbal IQ, processing speed and reading comprehension as covariates for ANOVAs. Pearson's correlation coefficients were calculated between the scores of all cognitive and mathematical tests. A series of linear hierarchical regression analyses were conducted to test the role of numerical magnitude processing (symbolic and non-symbolic numerical magnitude comparison) to arithmetic computation (simple and complex subtraction) of deaf adolescents, while controlling for demographic variables (i.e., age, gender; entering stage 1) and general cognitive abilities (i.e., non-verbal IQ, processing speed and reading comprehension; entering stage 2).

## Results

### Descriptive Statistics

The means and standard deviations and one-way analyses of variance of the scores for all seven tasks on the study groups are displayed in Table 1. We found a significant group effect on reading comprehension, arithmetic computation (simple and complex subtraction), symbolic magnitude comparison (accuracy and reaction time), and the accuracy of non-symbolic magnitude comparison but not on the reaction time of non-symbolic magnitude comparison. Hearing group outperformed congenital group, and congenital group outperformed acquired group in arithmetic computation and symbolic magnitude comparison (reaction time).

**Table 1:** All the measures on the study groups (M ± SD).

	**Index**	**A. Congenital group**	**B. Acquired group**	**C. Hearing group**	**Minimum**	**Maximum**	**Statistical difference**	
		**(*n* = 58)**	**(*n* = 54)**	**(*n* = 58)**			***F(2, 167)***	***LSD***
Age (months)		184.36 ± 28.12	188.44 ± 26.48	166.34 ± 21.75	98	231	12.03^***^	B, A > C
Non-verbal IQ	Adj. No. of correct response	12.36 ± 10.01	12.39 ± 10.71	12.79 ± 10.31	−12	30	0.03	—
PS. (ACC)	Accuracy (%)	93.55 ± 13.22	93.39 ± 13.38	95.73 ± 8.92	46	100	0.68	—
PS. (RT)	Reaction time (Millisecond)	483.56 ± 137.56	511.79 ± 135.35	411.49 ± 159.48	230.75	1232.50	7.23^**^	B, A > C
Reading Com.	Adj. No. of correct response	8.00 ± 10.83	4.46 ± 9.02	30.78 ± 8.48	−23	47	128.60^***^	B, A < C
Symbolic (ACC)	Accuracy (%)	87.41 ± 13.79	85.07 ± 16.82	93.72 ± 6.13	48	100	6.77^**^	B, A < C
Symbolic (RT)	Reaction time (Millisecond)	658.61 ± 145.92	719.95 ± 170.82	582.73 ± 120.24	393.00	1159.00	12.35^***^	B > A > C
Non-symbolic (ACC)	Accuracy (%)	68.00 ± 13.77	67.20 ± 15.30	74.38 ± 13.10	42	93	4.47^*^	B, A < C
Non-symbolic (RT)	Reaction time (Millisecond)	484.19 ± 133.38	533.10 ± 193.14	515.94 ± 136.16	232.00	1058.00	1.43	—
Simple subtraction	Adj. No. of correct response	27.97 ± 15.48	21.07 ± 14.52	41.94 ± 8.31	−5	59	37.06^***^	B < A < C
Complex subtraction	Adj. No. of correct response	10.67 ± 9.34	6.50 ± 9.78	19.31 ± 9.00	−19	33	27.46^***^	B < A < C

*Adj., adjusted; No., number; PS., Processing speed; Reading Com., Reading comprehension; ACC, accuracy; RT, reaction time*.

* *p < 0.05*,

** *p < 0.01*,

*** *p < 0.001*.

### Numerical Magnitude Comparison

**Figure 1:**
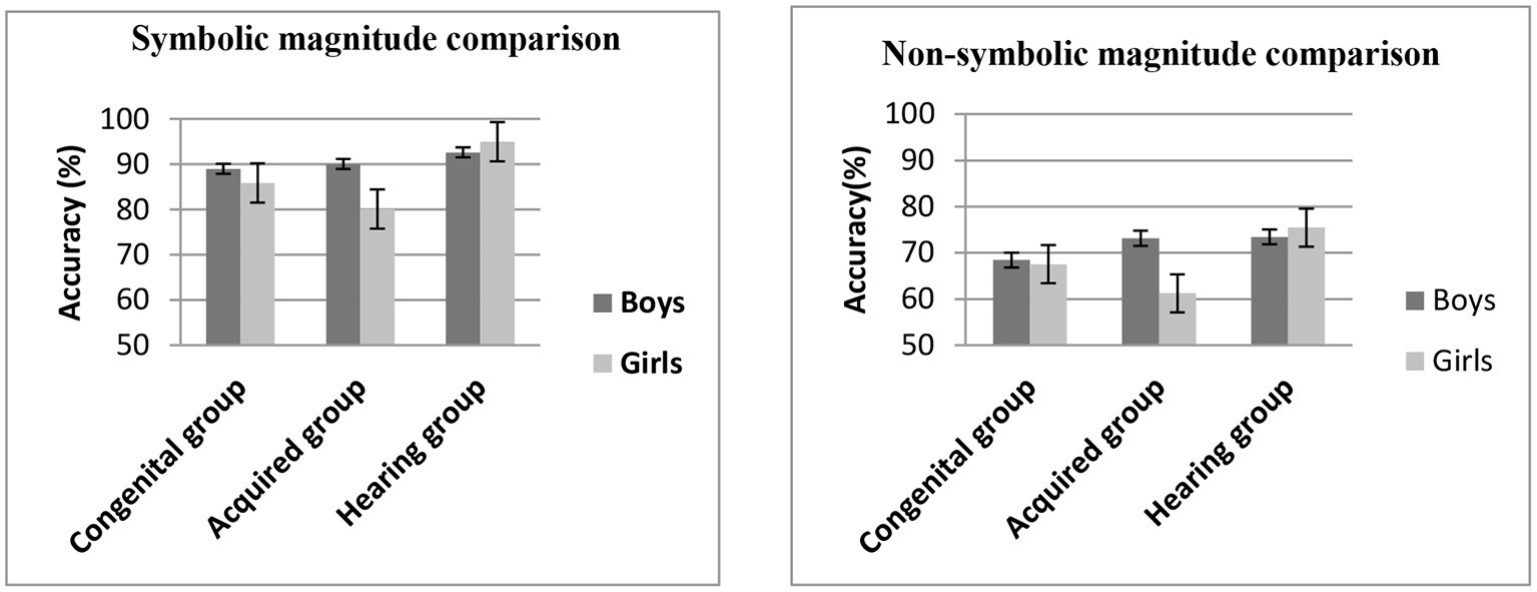
Mean accuracy (%) on the numerical magnitude comparison task (symbolic and non-symbolic) across groups.

A 2 × 2 ×3 mixed model, repeated measures ANOVA was conducted to examine whether the accuracy of numerical magnitude processing (symbolic, non-symbolic) varied by gender and group (see Figure 1). There was one within-subjects factor (numerical magnitude comparison: symbolic vs. non-symbolic) and two between-subjects factors: (gender: boys vs. girls) and (group: congenital, acquired, hearing). In order to control the effect of general cognitive abilities (e.g., reading comprehension) on numerical magnitude comparison, we used non-verbal IQ, processing speed and reading comprehension as covariates for ANOVA.

The main effect of gender, *F*_(1,160)_ = 5.26, η^2^ = 0.03, *p* < 0.05, was significant, indicating that the numerical magnitude processing of boys (81.00 ± 1.00%) was more accurate than that of girls (77.71 ± 1.02%). There was no a main effect of numerical magnitude comparison, *F*_(1,160)_ = 0.20, η^2^ = 0.001, *p* > 0.05, and there was no a main effect of group, *F*_(2,160)_ = 0.92, η^2^ = 0.011, *p* > 0.05; but the group × gender interaction was significant, *F*_(2,160)_ = 5.05, η^2^ = 0.06, *p* < 0.01. The simple effect test showed that for boys, there were no significant differences among the three groups (*p* > 0.05); for girls, there was no significant difference between congenital group and hearing group (*p* > 0.05), but the scores of acquired group were lower than those of hearing group significantly (*p* < 0.01) and congenital group marginally significantly (*p* = 0.056).

There were no significant two-way numerical magnitude comparison × group interaction, *F*_(2,160)_ = 0.56, η^2^ = 0.007, *p* = 0.57, and numerical magnitude comparison × gender interaction, *F*_(1,160)_ = 0.01, η^2^ = 0.000, *p* = 0.92. And there was no significant three-way numerical magnitude comparison × group × gender interaction, *F*_(2,160)_ = 0.17, η^2^ = 0.002, *p* = 0.84.

**Figure 2:**
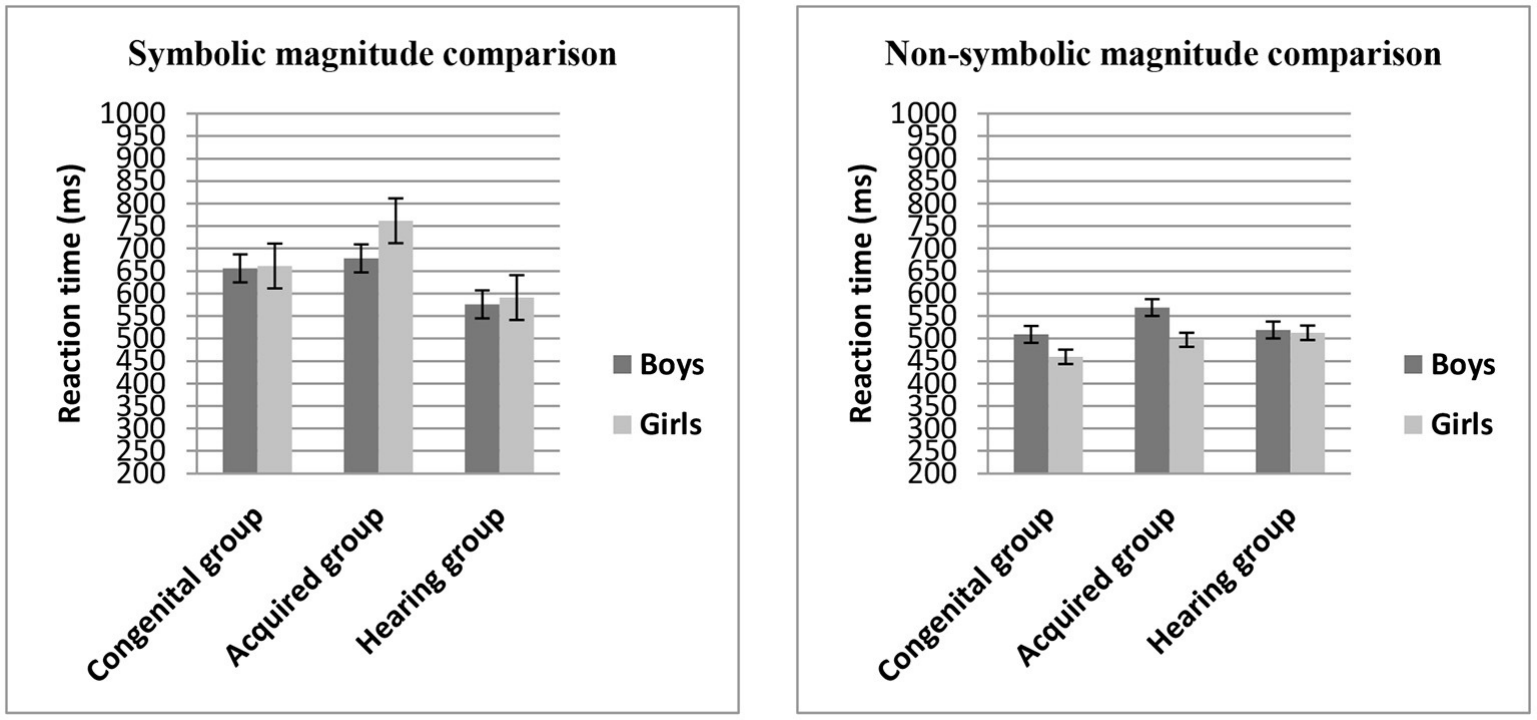
Mean reaction time (ms) on the numerical magnitude comparison task (symbolic and non-symbolic) across groups.

In order to examine whether the reaction time of numerical magnitude processing (symbolic, non-symbolic) varied by gender and group, a 2 × 2 × 3 mixed model, repeated measures ANOVA was again conducted with numerical magnitude comparison (symbolic vs. non-symbolic) as within-subject factor, gender (boys vs. girls), and group (congenital, acquired, hearing) as between-subject factors, and general cognitive abilities (non-verbal IQ, processing speed and reading comprehension) as covariates (see Figure 2).

The main effect of group, *F*_(2,160)_ = 3.42, η^2^ = 0.04, *p* < 0.05, was significant; LSD *post-hoc* comparisons showed that there was no significant difference in the reaction times between the congenital group and the hearing group (*p* > 0.05), but the reaction times of the acquired group were significantly longer than those of the congenital group and the hearing group (*p* < 0.05). There was no a main effect of numerical magnitude comparison, *F*_(1,160)_ = 0.57, η^2^ = 0.004, *p* > 0.05, and there was no a main effect of gender, *F*_(1,160)_ = 0.02, η^2^ = 0.000, *p* > 0.05; but the numerical magnitude comparison × gender interaction was significant, *F*_(1,160)_ = 7.29, η^2^ = 0.04, *p* < 0.01. The simple effect test showed that for boys and girls, the reaction time in symbolic magnitude comparison task was significantly longer than that in non-symbolic magnitude comparison task (*p* < 0.001).

There were no significant two-way numerical magnitude comparison × group interaction, *F*_(2,160)_ = 0.51, η^2^ = 0.006, *p* = 0.60, and group × gender interaction, *F*_(2,160)_ = 0.01, η^2^ = 0.000, *p* = 0.99. And there was no significant three-way numerical magnitude comparison × group × gender interaction, *F*_(2,160)_ = 1.32, η^2^ = 0.016, *p* = 0.27.

### Arithmetic Computation

**Figure 3:**
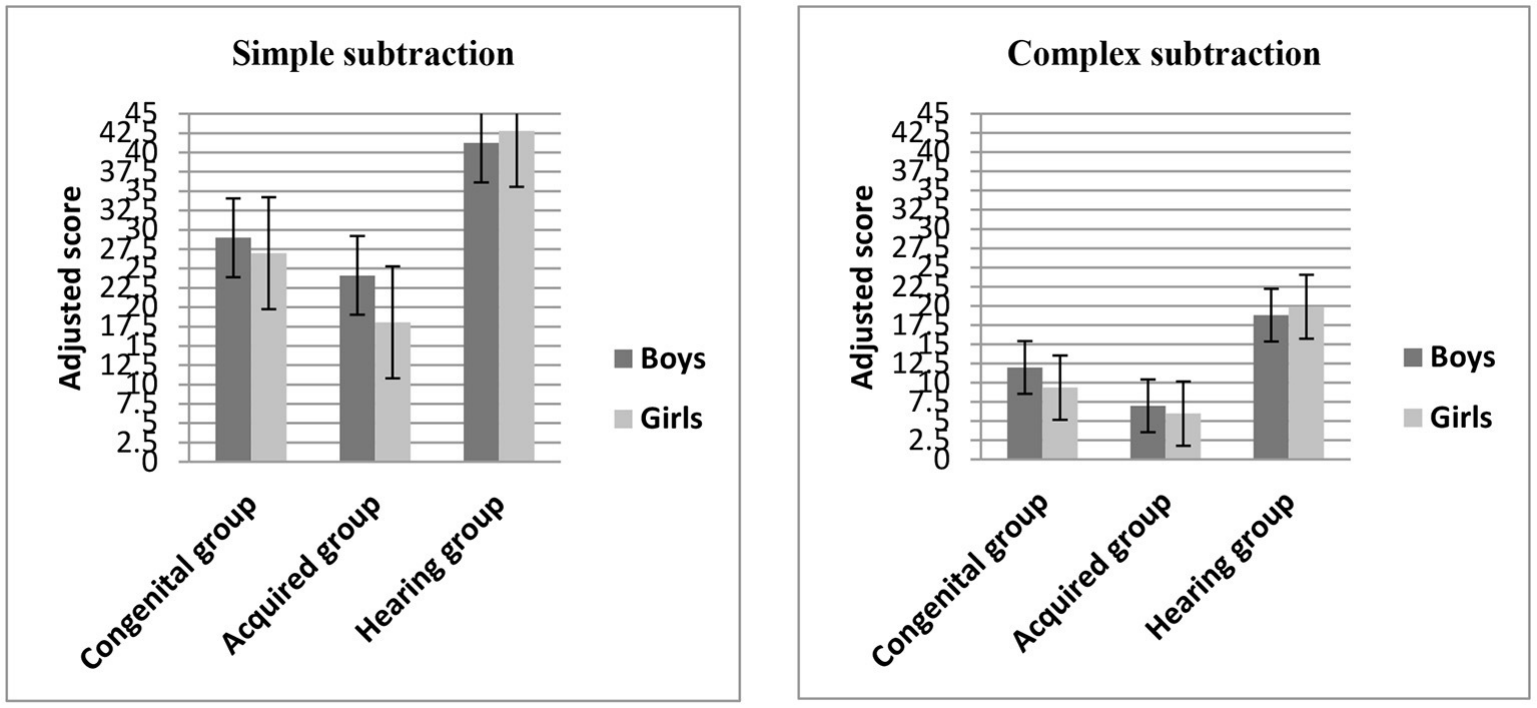
Mean adjusted scores on the arithmetic computation tasks (simple and complex subtraction) across groups.

To examine whether the performance of arithmetic computation (simple and complex subtraction) varied by gender and group, a 2 × 2 × 3 mixed model, repeated measures ANOVA was again conducted with arithmetic type (simple vs. complex) as within-subject factor, gender (boys vs. girls) and group (congenital, acquired, hearing) as between-subject factors, and general cognitive abilities (non-verbal IQ, processing speed, and reading comprehension) as covariates (see Figure 3).

The main effect of group, *F*_(2,160)_ = 3.32, η^2^ = 0.04, *p* < 0.05, was significant; LSD *post-hoc* comparisons showed that there were no significant differences in the scores between the congenital group and the hearing group (*p* > 0.05), but the scores of the acquired group were significantly lower than those of the congenital group and the hearing group (*p* < 0.05). There was no a main effect of arithmetic type, *F*_(1,160)_ = 0.06, η^2^ = 0.000, *p* > 0.05, and there was no a main effect of gender, *F*_(1,160)_ = 2.11, η^2^ = 0.013, *p* > 0.05.

And there were no significant two-way arithmetic type × group interaction, *F*_(2,160)_ = 0.47, η^2^ = 0.006, *p* > 0.05; arithmetic type × gender interaction, *F*_(1,160)_ = 1.61, η^2^ = 0.01, *p* > 0.05, and group × gender interaction, *F*_(2,160)_ = 0.40, η^2^ = 0.005, *p* > 0.05. And there was no significant three-way arithmetic type × group × gender interaction, *F*_(2,160)_ = 0.99, η^2^ = 0.012, *p* > 0.05.

### Numerical Magnitude Comparison and Arithmetic Computation

In order to explore the numerical magnitude processing in deaf adolescents and its contribution to arithmetical ability, we first analyze how numerical magnitude comparison and arithmetic computation may differ across groups and then consider the contribution of numerical magnitude processing to arithmetical ability within each group and in all deaf adolescents.

#### Analysis of Each Group of Deaf Adolescents

The partial correlations were separately calculated for each group between arithmetic computation (simple and complex subtraction), general cognitive abilities (i.e., non-verbal IQ, processing speed, and reading comprehension), and numerical magnitude processing (symbolic and non-symbolic) in deaf adolescents. A Bonferroni correction was used to maintain the *p*-value < 0.05 across the 45 correlations in Tables 2, 3. Thus, a conservative *p*-value of <0.00111 (=0.05/45) was considered statistically significant. As shown in Tables 2, 3, there was only a significant correlation between reading comprehension and simple subtraction in congenital group; However, there was a significant correlation between the accuracy of numerical magnitude processing (symbolic and non-symbolic) and simple subtraction and a significant correlation between the accuracy of non-symbolic numerical magnitude processing and complex subtraction, except for the significant correlation between reading comprehension and simple subtraction, in acquired group.

**Table 2:** Partial correlations after controlling for age and gender differences among all the test scores in congenital group.

	**1**	**2**	**3**	**4**	**5**	**6**	**7**	**8**	**9**	**10**
1 Non-verbal IQ	—									
2 PS. (ACC)	0.11	—								
3 PS. (RT)	−0.11	−0.29	—							
4 Reading Com.	0.35	0.07	−0.37	—						
5 Symbolic (ACC)	0.34	0.42^*^	−0.25	0.14	—					
6 Symbolic (RT)	−0.11	−0.13	0.33	−0.16	−0.08	—				
7 Non-symbolic (ACC)	0.15	0.11	−0.10	0.31	0.28	−0.37	—			
8 Non-symbolic (RT)	0.09	−0.09	−0.08	0.16	0.30	−0.08	0.73^*^	—		
9 Simple subtraction	0.29	0.26	−0.34	0.46^*^	0.30	−0.32	0.32	0.11	—	
10 Complex subtraction	0.21	0.24	−0.22	0.27	0.24	−0.31	0.30	0.12	0.66^*^	—

* *p < 0.05*,

*Bonferroni-corrected. PS., Processing speed; Reading Com., Reading comprehension; ACC, accuracy; RT, reaction time*.

**Table 3:** Partial correlations after controlling for age and gender differences among all the test scores in acquired group.

	**1**	**2**	**3**	**4**	**5**	**6**	**7**	**8**	**9**	**10**
1 Non-verbal IQ	—									
2 PS. (ACC)	0.37	—								
3 PS. (RT)	−0.22	−0.17	—							
4 Reading Com.	0.39	0.20	−0.35	—						
5 Symbolic (ACC)	0.35	0.26	−0.18	0.24	—					
6 Symbolic (RT)	−0.06	−0.15	0.44^*^	−0.20	−0.02	—				
7 Non-symbolic (ACC)	0.31	0.18	−0.39	0.31	0.31	−0.29	—			
8 Non-symbolic (RT)	0.21	0.21	0.00	0.15	0.10	0.33	0.53^*^	—		
9 Simple subtraction	0.30	0.17	−0.34	0.51^*^	0.51^*^	−0.37	0.48^*^	0.11	—	
10 Complex subtraction	0.25	−0.10	−0.27	0.34	0.25	−0.25	0.55^*^	0.31	0.56^*^	—

* *p < 0.05*,

*Bonferroni-corrected. PS., Processing speed; Reading Com., Reading comprehension; ACC, accuracy; RT, reaction time*.

A series of linear hierarchical regression analyses were conducted separately for each group to determine the contribution of numerical magnitude processing to the arithmetic ability (simple and complex subtraction) of deaf adolescents within each group. We also performed Bonferroni correction on the 2 regression analyses. Thus, a conservative *p*-value of <0.025 (=0.05/2) was considered statistically significant. According to Table 4, except that general cognitive abilities could account for 27.3% of the variation in simple subtraction [*F*_change (4,51)_ = 5.28, *p* = 0.001] and demographic variables could account for 14.1% of the variation in complex subtraction [*F*_change (2,55)_ = 4.51, *p* = 0.015], others did not have a contribution to the arithmetic ability of deaf adolescents in congenital group. However, general cognitive abilities [*F*_change (4,47)_ = 4.95, *p* = 0.002] could account for 27.9% and symbolic magnitude processing [*F*_change (2,45)_ = 8.98, *p* = 0.001] could account for 18.9% of the variation in simple subtraction; and general cognitive abilities [*F*_change (4,47)_ = 3.05, *p* = 0.026] could account for 19.6% and non-symbolic magnitude processing [*F*_change (2,43)_ = 7.12, *p* = 0.002] could account for 17.3% of the variation in complex subtraction in acquired group.

**Table 4:** Hierarchical regression models predicting arithmetic ability (simple and complex subtraction) from age, gender, general cognitive ability, symbolic, and non-symbolic magnitude processing in congenital and acquired group.

	**Simple subtraction** **β**				**Complex subtraction** **β**			
	**Step 1**	**Step2**	**Step3**	**Step4**	**Step 1**	**Step2**	**Step3**	**Step4**
**Congenital group**								
Age (months)	0.252	0.184	0.132	0.066	0.348^*^	0.297	0.234	0.173
Gender	−0.064	−0.096	−0.068	−0.093	−0.138	−0.154	−0.128	−0.141
Non-verbal IQ	—	0.136	0.079	0.081	—	0.117	0.070	0.077
Ps. (ACC)	—	0.176	0.120	0.084	—	0.173	0.130	0.115
Ps. (RT)	—	−0.147	−0.066	−0.105	—	−0.089	−0.003	−0.037
Reading Com.	—	0.333^*^	0.334^*^	0.292	—	0.164	0.164	0.118
Symbolic (ACC)	—	—	0.142	0.147	—	—	0.101	0.080
Symbolic (RT)	—	—	−0.210	−0.139	—	—	−0.247	−0.181
Non-symbolic (ACC)	—	—	—	0.229	—	—	—	0.197
Non-symbolic (RT)	—	—	—	−0.157	—	—	—	−0.073
	*R*^2^ = 0.068	*R*^2^ = 0.341*	*R*^2^ = 0.388	*R*^2^ = 0.401	*R*^2^ = 0.141*	*R*^2^ = 0.259	*R*^2^ = 0.312	*R*^2^ = 0.325
	(Δ*R*^2^ = 0.273*)(Δ*R*^2^ = 0.047)(Δ*R*^2^ = 0.013)				(Δ*R*^2^ = 0.118)(Δ*R*^2^ = 0.053)(Δ*R*^2^ = 0.012)			
**Acquired group**								
Age (months)	−0.117	−0.103	−0.216	−0.198	0.219	0.176	0.103	0.141
Gender	−0.198	−0.167	0.016	0.071	−0.077	−0.025	0.078	0.218
Non-verbal IQ	—	0.101	0.027	−0.005	—	0.207	0.178	0.111
Ps. (ACC)	—	0.028	−0.059	−0.062	—	−0.256	−0.303	−0.356^*^
Ps. (RT)	—	−0.170	−0.020	0.026	—	−0.177	−0.077	0.005
Reading Com.	—	0.389^*^	0.330^*^	0.310^*^	—	0.238	0.205	0.147
Symbolic (ACC)	—	—	0.428^*^	0.390^*^	—	—	0.201	0.157
Symbolic (RT)	—	—	−0.307^*^	−0.283	—	—	−0.218	−0.298
Non-symbolic (ACC)	—	—	—	0.209	—	—	—	0.277
Non-symbolic (RT)	—	—	—	0.022	—	—	—	0.279
	*R*^2^ = 0.058	*R*^2^ = 0.337*	*R*^2^ = 0.526*	*R*^2^ = 0.558	*R*^2^ = 0.050	*R*^2^ = 0.246*	*R*^2^ = 0.304	*R*^2^ = 0.477*
	(Δ*R*^2^ = 0.279*)(Δ*R*^2^ = 0.189*)(Δ*R*^2^ = 0.032)				(Δ*R*^2^ = 0.196*)(Δ*R*^2^ = 0.058)(Δ*R*^2^ = 0.173*)			

* *p < 0.05*,

*Bonferroni-corrected. Ps., Processing speed; Reading Com., Reading comprehension; ACC, accuracy; RT, reaction time*.

#### Analysis of All Deaf Adolescents

To examine the association between numerical magnitude processing and arithmetic ability in all deaf adolescents, partial correlations were computed and a Bonferroni correction was also used to maintain the *p*-value < 0.05 across the 45 correlations in Table 5. Thus, a conservative *p*-value of <0.00111 (=0.05/45) was considered statistically significant. As shown in Table 5, there was a significant correlation between deaf adolescents' reaction time on the symbolic magnitude comparison task and their performance on the arithmetic computation tasks (simple and complex subtraction), and there was also a significant correlation between deaf adolescents' accuracy on the non-symbolic magnitude comparison task and their performance on the arithmetic computation tasks (simple and complex subtraction).

**Table 5:** Partial correlations after controlling for age and gender differences among all the test scores.

	**1**	**2**	**3**	**4**	**5**	**6**	**7**	**8**	**9**	**10**
1 Non-verbal IQ	—									
2 PS. (ACC)	0.26	—								
3 PS. (RT)	−0.17	−0.23	—							
4 Reading Com.	0.36^*^	0.12	−0.37^*^	—						
5 Symbolic (ACC)	0.34^*^	0.34^*^	−0.24	0.20	—					
6 Symbolic (RT)	−0.08	−0.13	0.42^*^	−0.21	−0.08	—				
7 Non-symbolic (ACC)	0.27	0.17	−0.29	0.30^*^	0.31^*^	−0.34^*^	—			
8 Non-symbolic (RT)	0.18	0.10	−0.02	0.12	0.17	0.19	0.60^*^	—		
9 Simple subtraction	0.31^*^	0.23	−0.36^*^	0.49^*^	0.41^*^	−0.37^*^	0.42^*^	0.10	—	
10 Complex subtraction	0.23	0.07	−0.26	0.32^*^	0.25	−0.31^*^	0.42^*^	0.19	0.63^*^	—

* *p < 0.05*,

*Bonferroni-corrected. PS., Processing speed; Reading Com., Reading comprehension; ACC, accuracy; RT, reaction time*.

In order to determine the contribution of numerical magnitude processing to the arithmetic ability of all deaf adolescents, a series of linear hierarchical regression analyses were conducted. We also performed Bonferroni correction on the two regression analyses. Thus, a conservative *p*-value of <0.025 (=0.05/2) was considered statistically significant. According to Table 6, general cognitive abilities could account for 27.3% of the variation in simple subtraction [*F*_change (4,104)_ = 10.86, *p* < 0.001]. After controlling for scores of general cognitive ability and demographic variables, symbolic magnitude processing could account for 9.8% of the variation in simple subtraction [*F*_change (2,102)_ = 8.95, *p* < 0.001]. However, demographic variables [*R*^2^ = 0.072, *F*_change (3,108)_ = 2.81, *p* = 0.043, *p* > 0.025] and non-symbolic magnitude processing [*R*^2^ = 0.021, *F*_change (2,100)_ = 1.94, *p* = 0.149] did not have an additional contribution to simple subtraction.

**Table 6:** Hierarchical regression models predicting arithmetic ability (simple and complex subtraction) from age, gender, group, general cognitive ability, symbolic, and non-symbolic magnitude processing.

	**Simple subtraction**				**Complex subtraction**			
	**Step 1**	**Step2**	**Step3**	**Step4**	**Step 1**	**Step2**	**Step3**	**Step4**
	**β**	**β**	**β**	**β**	**β**	**β**	**β**	**β**
Age (months)	0.069	0.044	−0.025	−0.044	0.258^*^	0.219^*^	0.158	0.145
Gender	−0.133	−0.129	−0.040	−0.026	−0.105	−0.090	−0.025	0.014
Group(congenital/acquired)	−0.231^*^	−0.151	−0.093	−0.097	−0.234^*^	−0.186^*^	−0.138	−0.163
Non-verbal IQ	—	0.137	0.068	0.054	—	0.146	0.106	0.075
Ps. (ACC)	—	0.112	0.034	0.039	—	−0.029	−0.078	−0.079
Ps. (RT)	—	−0.174	−0.052	−0.043	—	−0.151	−0.051	−0.037
Reading Com.	—	0.335^*^	0.322^*^	0.297^*^	—	0.174	0.164	0.123
Symbolic (ACC)	—	—	0.280^*^	0.241^*^	—	—	0.165	0.110
Symbolic (RT)	—	—	−0.246^*^	−0.166	—	—	−0.223^*^	−0.175
Non-symbolic (ACC)	—	—	—	0.222	—	—	—	0.223
Non-symbolic (RT)	—	—	—	−0.076	—	—	—	0.068
	*R*^2^ = 0.072	*R*^2^ = 0.346*	*R*^2^ = 0.443*	*R*^2^ = 0.464	*R*^2^ = 0.121*	*R*^2^ = 0.231*	*R*^2^ = 0.284*	*R*^2^ = 0.339*
	(Δ*R*^2^ = 0.273*)(Δ*R*^2^ = 0.098*)(Δ*R*^2^ = 0.021)				(Δ*R*^2^ = 0.110*)(Δ*R*^2^ = 0.053*)(Δ*R*^2^ = 0.054*)			

* *p < 0.05*,

*Bonferroni-corrected. Ps., Processing speed; Reading Com., Reading comprehension; ACC, accuracy; RT, reaction time*.

Demographic variables (age, gender, onset of hearing loss) could account for 12.1% of the variation [*F*_change (3,108)_ = 4.97, *p* < 0.01], and general cognitive abilities could account for 11.0% of the variation [*F*_change (4,104)_ = 3.71, *p* < 0.01] in complex subtraction. After controlling for scores of general cognitive abilities and demographic variables, symbolic magnitude processing could account for 5.3% of the variation [*F*_change (2,102)_ = 3.81, *p* < 0.025] and non-symbolic magnitude processing could account for 5.4% of the variation [*F*_change (2,100)_ = 4.11, *p* < 0.025] in complex subtraction.

## Discussion

The current study aimed to examine numerical magnitude processing and its contribution to arithmetical ability in deaf adolescents. The main results are summarized as follows: First, repeated measures ANOVA showed that the numerical magnitude processing of boys was more accurate than that of girls. For boys, there were no significant differences among the three groups (congenital, acquired, and hearing) in the accuracy of numerical magnitude processing; for girls, there was no significant difference between congenital group and hearing group, but the accuracy in acquired group was lower than that in hearing and congenital group significantly. Second, one-way ANOVA showed hearing adolescents outperformed deaf adolescents in arithmetic computation (simple and complex subtraction), symbolic magnitude processing (accuracy and reaction time), and the accuracy, but not the reaction time of non-symbolic magnitude processing. Third, the hierarchical regression analyses of each group of deaf adolescents showed that numerical magnitude processing did not have a contribution to arithmetic computation in congenital group, but symbolic magnitude processing could contribute to simple subtraction and non-symbolic magnitude processing could contribute to complex subtraction in acquired group.

### Numerical Magnitude Processing and Arithmetic Ability in Deaf Adolescents

The results of one-way ANOVA showed that deaf adolescents lag behind hearing adolescents in arithmetic computation (simple and complex subtraction), symbolic magnitude processing (accuracy and reaction time), and the accuracy, but not the reaction time of non-symbolic magnitude processing. It is basically consistent with the previous results (e.g., Rodríguez-Santos et al., 2014; Masataka, 2006) that deaf individuals were found worse performance on symbolic but not non-symbolic magnitude processing, indicating the delay of deaf individuals in symbolic but not non-symbolic encoding. According to “access deficit hypothesis” (Rouselle and Noël, 2007), deficits in the representation of numerical information in long-term memory are not general, but are linked to the numerical representation codes used for its acquisition (Arabic numerals, number words). Deaf individuals' poor performance on an Arabic number comparison task, but not on a dot collection comparison task, could be explained by difficulties in accessing the semantic information of numbers by means of symbols, due to their low-level language and their limited experience with numbers (Gregory, 1998; Nunes, 2004; Kritzer, 2009; Bull et al., 2011).

It was also found that boys outperformed girls in the accuracy of numerical magnitude processing in the study. The result was similar to the previous study of Krinzinger et al. (2012), but scare previous researches on gender differences in numerical magnitude processing in deaf individuals. Krinzinger et al. (2012) applied structural equation modeling to a longitudinal dataset of 140 primary school children and found superiority for primary school boys in numerical magnitude processing. One explanation of Krinzinger et al.'s results is that general visual-spatial abilities (but not visual-spatial working memory), which has been found to favor males (Goldstein et al., 1990; Vederhus and Krekling, 1996).

And hierarchical regression analyses of all deaf adolescents showed that numerical magnitude processing had an independent contribution to arithmetic computation after controlling for general cognitive ability. Previous studies have shown that the understanding of numerical magnitudes is helpful to the solution of arithmetic problems (De Smedt et al., 2009; Tavakoli, 2016). Neuroimaging studies have also revealed that numerical magnitude processing is related to arithmetic problem solving (Bugden et al., 2012; Price et al., 2013). It was also found the close relationship between the approximate number system acuity (non-symbolic numerical magnitude processing) and math achievement in children with hearing loss in the research of Bull et al. (2018), which is basically consistent with the results of this study.

### Onset of Hearing Loss (Congenital vs. Acquired) and Mathematical Cognition of Deaf Adolescents

Acquired deafness, as the type of deafness occurring after the acquisition of speech (Hindley and Kitson, 2000), is the loss of hearing that occurs after birth and develops sometimes during a person's life. Congenital deafness, in which auditory system has not been programmed for language and communication, is the loss of hearing that was present at birth. Although the difference between congenital deafness and acquired deafness is obvious, there are few studies on the difference between them in academic achievement such as mathematics performance. DeLeon et al. (1979) explored the reading and math skills of two groups of adults either congenital or acquired deafness matched in intelligence, education level and degree of loss, and found no significant differences on reading level between the two groups, but a significantly higher math level in the congenital group than the acquired. But in the research of Ogundiran and Olaosun (2013), no significant differences were found in the academic achievement including mathematics and English Language performance between students with congenital deafness and those with acquired deafness. And the results of our study that there was no significant difference between the congenital group and the hearing group, but congenital group outperformed acquired group in numerical magnitude processing (RT) and arithmetic computation, suggesting that the mathematical cognitive abilities of the congenital deaf are better than those of the acquired deaf, which is basically consistent with the results of DeLeon et al. (1979).

Compensatory plasticity holds that the lack of auditory stimulation experienced by deaf individuals, such as congenital deafness, can be met by enhancements in visual cognition (Neville, 1990; Bavelier et al., 2006). Previous studies have shown that auditory deprivation, such as congenital deafness, can lead to enhanced peripheral visual processing, which should be contributed by the neuroplasticity in multiple systems including primary auditory cortex, supramodal, and multisensory regions (Bavelier and Neville, 2002; Scott et al., 2014). According to the connectome model, congenital sensory loss, such as congenital deafness, is thought to be a connectome disease. It is an abnormal bias in the individual wiring and coupling pattern of the brain, which might result in stronger coupling to the remaining sensory systems and reorganization within the affected sensory system. This process accounts for the abnormal visual dominance in perception after congenital deafness (Kral et al., 2016).

Although some studies have found that the processing of sign language in the brain network of congenitally deaf individuals who acquired sign language from birth from their deaf parents is similar to that for spoken words in hearing individuals. The activity in their language network is due to a kind of semantic encoding rather than visual processing (Leonard et al., 2012). The electrophysiological study of congenitally deaf adolescents revealed that better visual processing could be explained by the early latency in N1 component in visual related brain responses associated with more efficient neural processing due to auditory deprivation (Güdücü et al., 2019). And the studies of hearing individuals also showed that visual form perception had unique contributions to lower level math categories, such as numerosity comparison, digit comparison, and exact computation (Cui et al., 2017); and it was the cognitive mechanism that could explain the association between numerical magnitude processing (e.g., approximate number system) and arithmetic computation (Zhang et al., 2019).

Therefore, it may be due to the advantages of visual processing, congenitally deaf individuals outperformed acquired deafness in mathematics. And according to the TCM and related neuropsychological researches, patients (with impaired auditory speech representation) could perform non-verbal numerical magnitude processing that manipulates analog magnitude representations by manipulating visual Arabic representations (Cipolotti and Butterworth, 1995; Cohen et al., 2000). Compared to the acquired deafness, individuals with congenital deafness may be more dependent on this non-verbal, visual representation due to auditory deprivation. It is also possible because that there is only visual processing (representation) in congenital deafness, but the conversion of auditory speech and visual representation/coding is needed in acquired deafness, which may lead to the hindrance of processing.

### Practical Implications

The current study offers several important insights and practical implications. First, since we found deaf adolescents lag behind hearing peers in symbolic but not non-symbolic magnitude processing, and symbolic magnitude processing accounted for unique variance in children's mathematical achievement (De Smedt et al., 2009; Bugden and Ansari, 2011), this suggests that educators should place great emphasis on helping their deaf students to understand the meaning of numerical symbols, thereby enhancing their ability to map number symbols unto non-symbolic quantities. Learning to accurately map symbolic magnitudes onto non-symbolic magnitudes is a crucial step toward performing more complex mathematics such as arithmetic operations (Siegler and Booth, 2004; Booth and Siegler, 2008; Geary et al., 2008). Second, we found general cognitive abilities (i.e., non-verbal IQ, processing speed and reading comprehension) could account for unique variance in deaf adolescents' arithmetic computation (simple and complex subtraction), which shows that the general cognitive abilities are the important influencing factors for the arithmetical ability in deaf adolescents. According to the developmental model of numerical cognition (von Aster and Shalev, 2007), the development of mathematical abilities in children is based on general cognitive abilities. Therefore, parents and teachers should promote the development of general cognitive abilities, such as intelligence, processing speed, and reading comprehension, in deaf children through activities and training as soon as possible, so as to improve their mathematics performance.

### Limitations and Prospects

There are some limitations to our work. First, the sample size was limited, only 112 deaf adolescents but not young deaf children were included in this study. Second, the test of arithmetic ability only examined by simple and complex subtraction, other tests such as simple and complex addition were not included. Third, reading comprehension was only regarded as a control variable, and other language abilities were not evaluated in the present study. The neural mechanism of congenital deafness in mathematical ability should be further investigated across the groups of congenital and acquired deafness and hearing counterparts.

## Conclusions

Consistent with the previous results, the study shows the worse performance on symbolic but not non-symbolic magnitude processing in deaf adolescents, which indicates that the lag of mathematics in deaf individuals may be due to the delay of symbolic but not non-symbolic encoding. It was found that boys outperformed girls in the accuracy of numerical magnitude processing in the study. Based on previous studies, it may be that the superiority of male visual-spatial ability improves their numerical magnitude processing. There was no significant difference between the congenital group and the hearing group, but congenital group outperformed acquired group in numerical magnitude processing (RT) and arithmetic computation. Similarly, it may be due to the advantage of visual processing that congenitally deaf individuals outperformed acquired deafness in mathematics. It was also found a close association between numerical magnitude processing and arithmetic computation of deaf adolescents, and after controlling for the demographic variables (age, gender, onset of hearing loss) and general cognitive ability (non-verbal IQ, processing speed, reading comprehension), numerical magnitude processing could predict arithmetic computation in all deaf adolescents but not in congenital group. The role of numerical magnitude processing (symbolic and non-symbolic) in deaf adolescents' mathematical performance should be paid attention in the training of arithmetical ability.

## Data Availability Statement

The raw data supporting the conclusions of this article will be made available by the authors, without undue reservation.

## Ethics Statement

The studies involving human participants were reviewed and approved by the Ethics Committee of Hainan Normal University. Written informed consent to participate in this study was provided by the participants' legal guardian/next of kin.

## Author Contributions

LC designed the study, carried out the experiment, analyzed the data, and wrote the manuscript. YW supervised part of the work. HW edited the manuscript. All authors contributed to the article and approved the submitted version.

## Conflict of Interest

The authors declare that the research was conducted in the absence of any commercial or financial relationships that could be construed as a potential conflict of interest.
